# Green synthesis of *in situ* electrodeposited rGO/MnO_2_ nanocomposite for high energy density supercapacitors

**DOI:** 10.1038/srep16195

**Published:** 2015-11-05

**Authors:** Rusi, S. R. Majid

**Affiliations:** 1 Centre for Ionics University of Malaya, Department of Physics, Faculty of Science, University of Malaya, 50603 Kuala Lumpur, Malaysia

## Abstract

This paper presents the preparation of *in situ* electrodeposited rGO/MnO_2_ nanocomposite as a binder-free electrode for supercapacitor application. The work describes and evaluates the performance of prepared electrode via green and facile electrodeposition technique of *in situ* rGO/MnO_2_-glucose carbon nanocomposites. The carbon content in the composite electrode increased after GO and D (+) glucose solution has been added in the deposition electrolyte. This study found that a suitable concentration of D (+) glucose in the deposition electrolyte can slow down the nucleation process of MnO_2_ particles and lead to uniform and ultrathin nanoflakes structure. The optimize electrode exhibited low transfer resistance and resulted on excellent electrochemical performance in three electrolyte systems viz. Na_2_SO_4_, KOH and KOH/K_3_Fe(CN)_6_ redox electrolytes. The optimum energy density and power density were 1851 Whkg^**−**1^ and 68 kWkg^**−**1^ at current density of 20 Ag^**−**1^ in mixed KOH/K_3_Fe(CN)_6_ electrolyte.

Nowadays, the need and use of energy is one of the necessities on diverse scale of modern technology. However, the increasing demand on energy leads to environmental issues and depleting fossil fuels Therefore, the intense research on energy storage and conversion has attracted much attention for future technology development. Supercapacitors have attracted growing interest, due to their high power density, long cycle life, and fast charging rate, which is playing an important role in complimenting or even replacing batteries in many applications^1^,^2^,^3^. Nevertheless, the low-energy density and higher production cost are still some of the major challenges for implementing supercapacitor in future application. To date, the carbon materials (activated carbon, carbon nanotubes, (CNT) and reduced graphene oxide (rGO))^1^,^2^,^4^, transition metal oxides (ruthenium dioxide (RuO_2_), manganese dioxide (MnO_2_), nickel oxide (NiO), cobalt oxide (Co_3_O_4_))^2^,^3^,^5^ and conducting polymers (polypyrrole, polyanaline, PEDOT-PSS and polythiophene)^6^,^7^ have been recognized as the most promising materials for supercapacitors. Based on literature study, the carbon-based electrodes display an excellent rate of capability, good reversibility, and superior cyclability but suffer from low capacitance value^2^. On the other hand, transition metal oxides and polymer-based electrodes produce high capacitance through a fast faradic reaction but have a poor rate of capability and stability^1^,^8^. Therefore, hybrid electrode materials, such as carbon-metal oxide-based electrodes, have become necessary for producing high capacitive performance and good cyclability.

Among the transition metal oxides, MnO_2_ has attracted more attention as a pseudocapacitor electrode material and has been widely studied due to its high theoretical capacitance (1370 Fg^**−**1^), natural abundance, environmental compatibility and low cost^4^,^9^,^10^. However, MnO_2_ has a low specific surface area and poor electrical conductivity (10^**−**5^ to10^**−**6^ Scm^**−**1^) associated with slow redox reaction kinetics, which often limits supercapacitor application^2^,^3^,^11^. In order to improve the electrical conductivity of MnO_2_ electrodes, the incorporation of highly conductive secondary materials to form hybrid compounds is being investigated. Graphene oxide holds great potential to be coupled with MnO_2,_ because it has high conductivity, good chemical stability, and a large surface area. In addition, the surfaces of graphene are capable of a reversible pseudo-reaction and electrochemical double layer formation, which is beneficial to the electrochemical performance of MnO_2_/graphene oxide composite materials^1^,^8^,^12^,^13^.

MnO_2_/graphene oxide composite electrode material synthesized by a hydrothermal method at various reaction times exhibited an optimized specific capacitance (SC) of 213 Fg^**−**1^ at current density of 0.1 Ag^**−**1^ 13. Under the same method, Deng *et al.* prepared MnO_2_ nanorods/graphene composites and demonstrated that the composites ratio of MnO_2_ on the graphene sheets is very important in order to obtain good electrochemical performance of the supercapacitor materials. The achieved specific capacitance is 218 Fg^**−**1^ and was determined by a cyclic voltammetry method at a scan rate of 5 mVs^**−**1^ in 1 M Na_2_SO_4_ aqueous solution^14^. In the work of Sawangphruk *et al.*, MnO_2_-rGO nanocomposites on graphitized carbon fiber paper was produced by a simple spray coating technique and exhibited a SC of 393 Fg^−1^ determined by a cyclic voltammetry method at a scan rate of 10 mVs^**−**1^in 0.5 M Na_2_SO_4_. This high capacitance value is most likely attributed to the synergistic effect originating from the high surface area of MnO_2_ nanoparticles, the high conductivity of rGO nanosheets, and the high porosity of MnO_2_-rGO nanosheets coated on CFP^12^. The composite electrode consisting of rGO and MnO_2_ nanoneedles prepared through hydrazine hydrate-mediated reduction of graphene oxide (rGO)/MnO_2_ displayed the highest SC among the composite electrodes studied with a SC as high as 371.74 Fg^−1^ at a scan rate of 10 mVs^**−**1^. Kim *et al.* have validated the experimental results of the synergistic effects of the EDL capacity, the excellent electrical conductivity of graphene oxide, and the homogeneously dispersed MnO_2_ nanoneedles^15^. A more green approach of reducing graphene nanosheet/urchin-like MnO_2_ composite preparation for a supercapacitor electrode uses the glucose molecule as a reductant and the oxidized product is environmentally friendly. The obtained electrode exhibited a SC of 263 Fg^**−**1^ in a 1.0 M Na_2_SO_4_ electrolyte solution calculated from constant current charge/discharge curves at 5 mAcm^−2^. The excellent interfacial contact between MnO_2_ and graphene is believed to have contributed to the fast transportation of electrons throughout the whole electrode matrix^1^.

Herein, we report a facile and green method to prepare reduced graphene oxide/manganese dioxide (rGO/MnO_2_) with a glucose carbon composite (i.e., an *in situ* electrodeposition technique). The prepared electrode was heated to exceed the decomposition temperature of glucose with the hope that the presence of carbon from glucose in the electrode would increase the effectiveness of cation pathways from electrolyte to electrode. The schematic presentation of *in situ* electrodeposition of MnO_2_-rGO with and without glucose on stainless steel is shown in Fig. 1.

The dispersed GO in water is negatively charged due to some ionization of carboxyl and hydroxyl functional groups on the GO surface^16^. Those functional groups act as anchor sites, which allow the positive-charged ions to be absorbed on the surface and edge of the negatively-charged GO sheets^17^. When the Mn(CH_3_COO)_2_·4H_2_O was added to the GO suspension solution, the Mn^2+^ ion was bonded with the oxygen atom of the negatively-charged residual oxygen-containing functional groups on the graphene oxide via the electrostatic force. Then, upon electrodeposition, the GO sheets with absorbed Mn^2+^ ions were deposited together to form manganese hydroxide and were reduced to graphene oxide. It is believed that adding glucose causes some molecules of Mn^2+^ will bind together with the hydroxyl group of glucose via electrostatic interaction and will deposit together in the SS. Manganese hydroxide will be converted to manganese oxide after being heated at 300 °C for 6 h.

## Results

**Figure 2:**
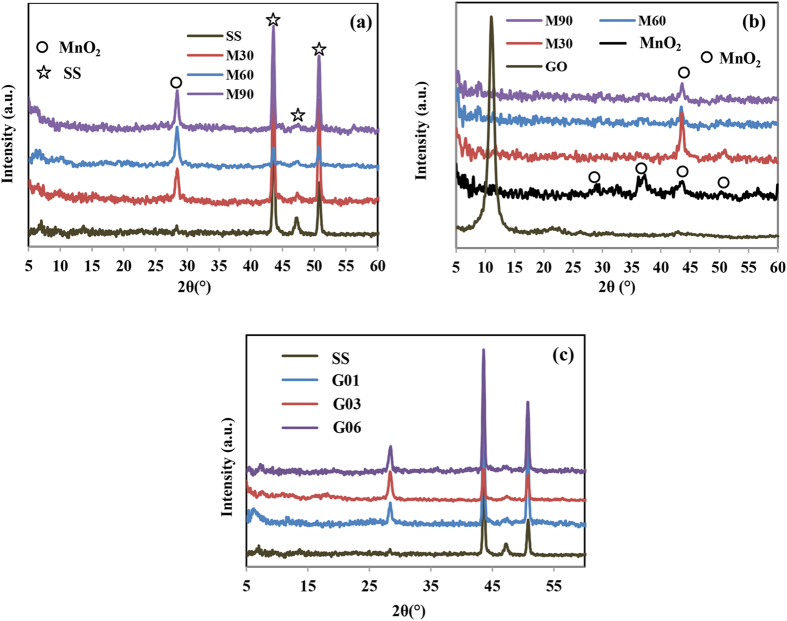
XRD pattern of: (**a**) M30, M60, and M90 on top of SS, (**b**) scraped-off deposits powder of M30, M60, and M90, (**c**) G03, G06, and G09 on top of SS.

In this study, we performed an *in situ* electrodeposition of manganese hydroxide, graphene oxide, and glucose to yield rGO/MnO_2_ nanocomposite electrode materials after heat treatment at 300 °C. The XRD patterns of as-prepared rGO-MnO_2_ deposits with and without glucose on top of SS and pure SS as references are shown in Fig. 2. Compared to the XRD pattern of stainless steel, there is an additional peak at 2θ = 28.3° in the diffractograms of deposited M30, M60, and M90 electrodes, Fig. 2a. This peak is attributable to the (310) plane of MnO_2_ and the intensity of the peak increased as the content of manganese ion in the deposition electrolyte increased. For clearer evidence of deposited rGO-MnO_2_, the XRD diffractogram for scraped-off deposits powder of M30, M60, and M90 was carried out as shown in Fig. 2b. The characteristic peaks of MnO_2_ observed at 2θ = 28.7°, 36.9°, 42.9°, and 50.3° are attributed to the (310), (211), (301) and (411) planes, which can be indexed to a tetragonal phase of α-MnO_2_ with lattice constant a = 9.7847 Å, c = 2.8630 Å (JCPDS 44–0141)^15^,^18^. In all XRD patterns of rGO-MnO_2_ scraped-off powders, an appreciable peak is observed at 2θ = 42.9°, which is associated with the (301) plane of MnO_2_. The peaks of GO in the deposit powders of the M30, M60, and M90 electrodes are hardly noticeable, suggesting that reduction of GO has taken place^19^,^20^. Figure 2c shows the XRD patterns of deposited rGO-MnO_2_ on top of SS with the presence of different concentrations of glucose molecules in the deposition electrolyte. The XRD results show that the addition of glucose molecules did not change the structure of the sample, since only one peak is observable that centred at 28.3°, which is attributed to the (310) plane of MnO_2_.

**Figure 3:**
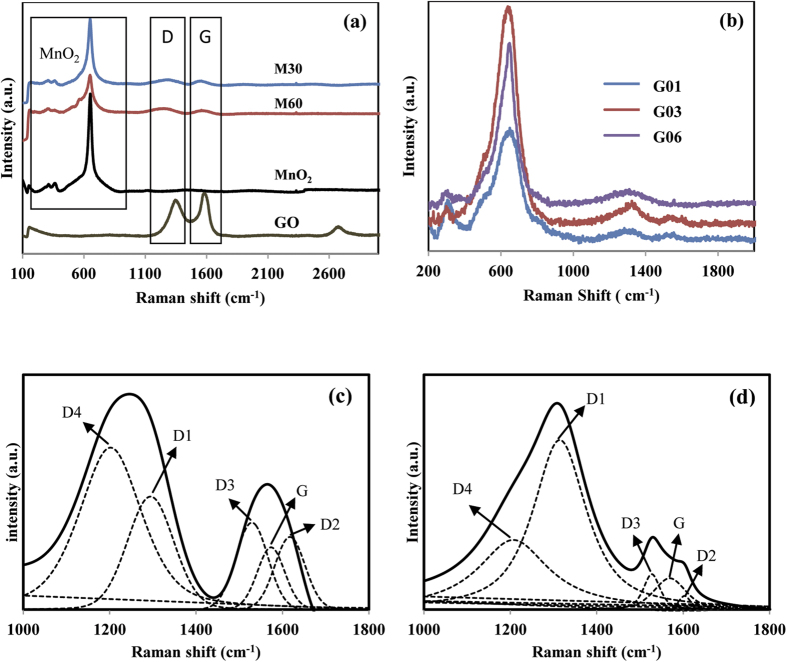
(**a**) Raman spectroscopy of deposited rGO-MnO_2_ electrode without glucose in electrolyte, (**b**) Raman spectroscopy of deposits rGO-MnO_2_ electrode with glucose in electrolyte, (**c**) Deconvolution of Raman spectra for M30 in the range of 1000 to 1800 cm^**−**1^, (**d**) Deconvolution of Raman spectra for G03 in the range of 1000 to 1800 cm^**−**1^.

To study the effect of glucose on the deposited rGO-MnO_2_ in more detail, Raman microscopy was performed. Figure 3a,b displays the Raman spectrum of as-heated rGO/MnO_2_ with and without the addition of glucose in deposition electrolytes. The characteristics peaks of GO centered at 1358 cm^−1^, 1579 cm^−1^, and 2675 cm^−1^ were attributed to D-band, G-band, and 2D-band, respectively. The D-band is related to the vacancies, edge defects, grain boundaries, and disordered carbon species in graphite layers, and G-band is due to the vibration of sp^2^ hybridized C-C bonds in two dimensional hexagonal lattice^20^,^21^,^22^. The intensity ratio of the D and G-bands (I_D_/I_G_) can be used to evaluate the sp^2^ domain size of a carbon structure and partially ordered crystal structure of graphenes^23^. The I_D_/I_G_ ratio of GO powder is 0.78 and the M30 and M60 electrodes resulted in increments of the I_D_/I_G_ ratio to 2.37 and 1.78 (Fig. 3a), which can be attributed to an increase in defects on the surface of the reduced GO that were induced during the synthesis process. Furthermore, the G band and D band in the prepared electrode got shifted to lower wavenumbers of around 1242 cm^**−**1^ and 1568 cm^**−**1^ for the M30 and M60 electrode, revealing that rGO are deposited^24^. The addition of glucose content in the deposition electrolyte has resulted in an increase of D band intensity (Fig. 3b), indicating an increase of disorder carbon in the graphite layers. The increment of D peak intensity might be attributed to the bands combination of D1, D2, D3 and D4 in the region from 1000 to 1800 cm^**−**1^, thus the deconvoluted of Raman spectra in this region for selected samples M30 and G03 is displayed in Fig. 3c,d. The deconvolution results for both samples clearly showed that the peak at around 1560–1598 cm^**−**1^ is related to the G peak. Peaks of D1 and D2 can be observed at around 1301–1317 cm^**−**1^ and 1599–1624 cm^**−**1^, respectively. Depiction of another two peaks at around 1489–1545 cm^**−**1^ and 1127–1200 cm^**−**1^ are corresponded to D3 and D4 peaks. The I_D_/I_G_ ratio (area) and vibration mode^25^ of M30 and G03 are listed in Table 1. The increment of the D band intensity mainly arises from overlapping of D1 and D4 peaks in the band region of 1100–1400 cm^**−**1^, suggesting that the disordered carbon in graphitic lattice has increased. In all deposited samples, the presence of a sharp peak at 650 cm^**−**1^ corresponds to MnO_2_ that is attributed to stretching vibrations of MnO_6_ octahedral^20^.

**Figure 4:**
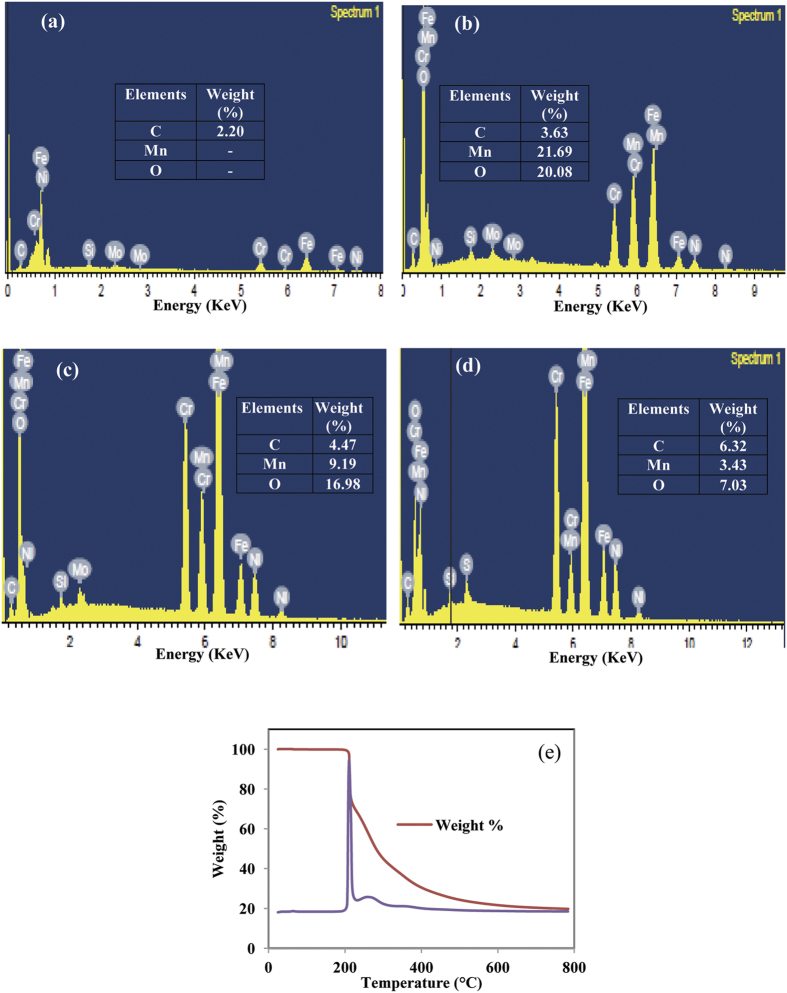
EDX spectrum of: (**a**) empty SS, (**b**) MnO_2_ without GO, (**c**) M30, (**d**) G03, and (**e**) TGA analysis of D (+) glucose.

The elements of deposits on top of SS were also studied using EDX spectrums, the pristine MnO_2_, M30, and G03 electrodes are shown in Fig. 4. The presence of Cr, Fe, Ni, Si, and Mo belongs to SS (Fig. 4a) and the presence of new elements such as Mn and O is assigned to formation MnO_2_ in electrode. The increment of carbon weight percentage in the M30 and G03 electrodes can be seen in Fig. 4c,d. The increment of carbon content in the M30 electrode is contributed from deposited of rGO carbon, while the G03 electrode is from rGO carbon and glucose decompose carbon. Glucose is considered to be the one of the most organic compounds that will decompose to a carbon solid element (known as carbon sources)^26^. Figure 4e displays the TGA analysis of D(+) glucose, in which the decomposition temperature of glucose was found to be around 250 °C. The heating temperature of our deposited electrodes was 300 °C, which exceeds the glucose decomposition temperature. Therefore, the increase of carbon content of the G03 electrode in the Raman and EDX analyses is believed from carbon of glucose decompose.

**Figure 5:**
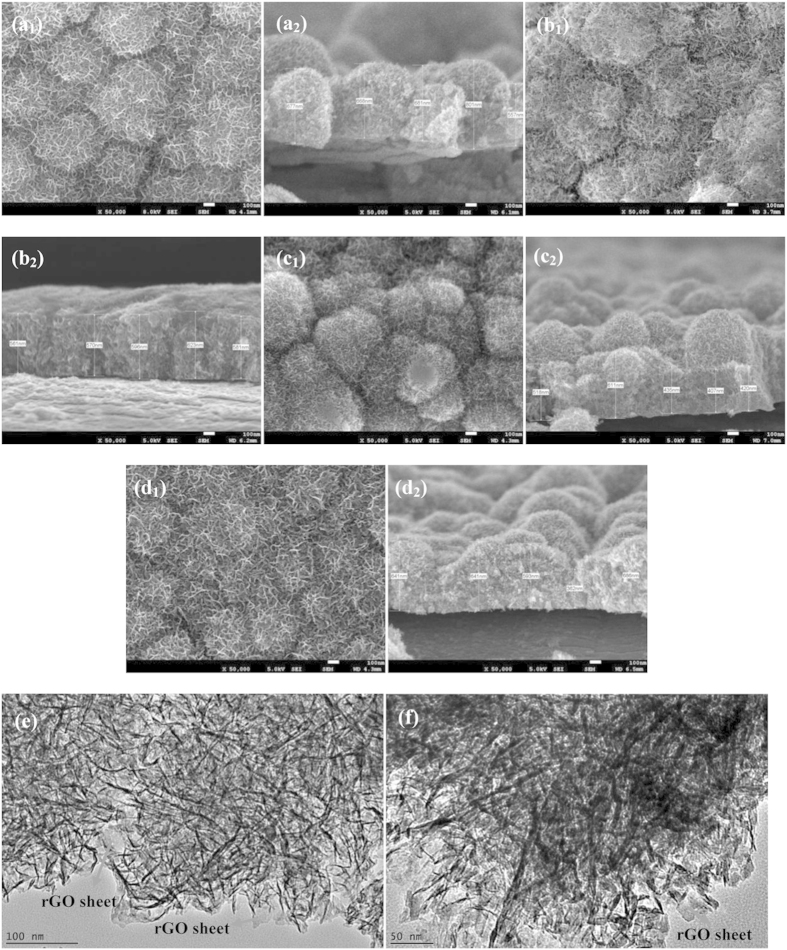
FESEM morphology images and cross section thickness of: (**a_1_, a_2_**) pristine MnO_2_, (**b_1_, b_2_**) M30, (**c_1_, c_2_**) M60, and (**d_1_, d_2_**) M90; TEM images of: (**e**) M30 and (**f**) M60.

FESEM was carried out to characterize the morphology structures of as-heated rGO/MnO_2_ deposited from different deposition electrolyte contents as shown in Fig. 5. The FESEM image of GO-free deposited MnO_2_ (as a control sample) exhibited round shape particles covered with nanoflake-like structures on the top. The thickness of the deposited particles is in the range of 550 to 801 nm, Fig. 5a_2_. When GO was added into the deposition electrolyte, M30 electrode grew with less flake-like structures, which may due to the slow nucleation process of MnO_2_ flakes and caused by the presence of rGO sheets, Fig. 5b_1_. The cross-section of the M30 electrode in Fig. 5b_2_ indicate less agglomerated MnO_2_ and more uniform thickness within a range from 560 to 630 nm. Further increases of Mn ion in deposition electrolyte, the structure of M60 and M90 electrodes almost looked like pristine MnO_2_ structure, Fig. 5c_1_,d_1_. The size of deposited electrode is thicker as observed in the cross-section images Fig. 5d_2_. The thick, dense structure generally can limit the diffusion of electrolyte cations toward the entangled oxide, resulting in low utilization of MnO_2_^27^. The morphological studies of M30 and M60 deposits were further investigated by using high magnification TEM, Fig. 5e,f. The MnO_2_ has dispersed uniformly on the rGO sheets. The TEM image of the M30 electrode displayed an rGO sheet with a thin flakes structure, whereas the M60 electrode showed thicker MnO_2_ flakes, which is in agreement with the FESEM result.

**Figure 6:**
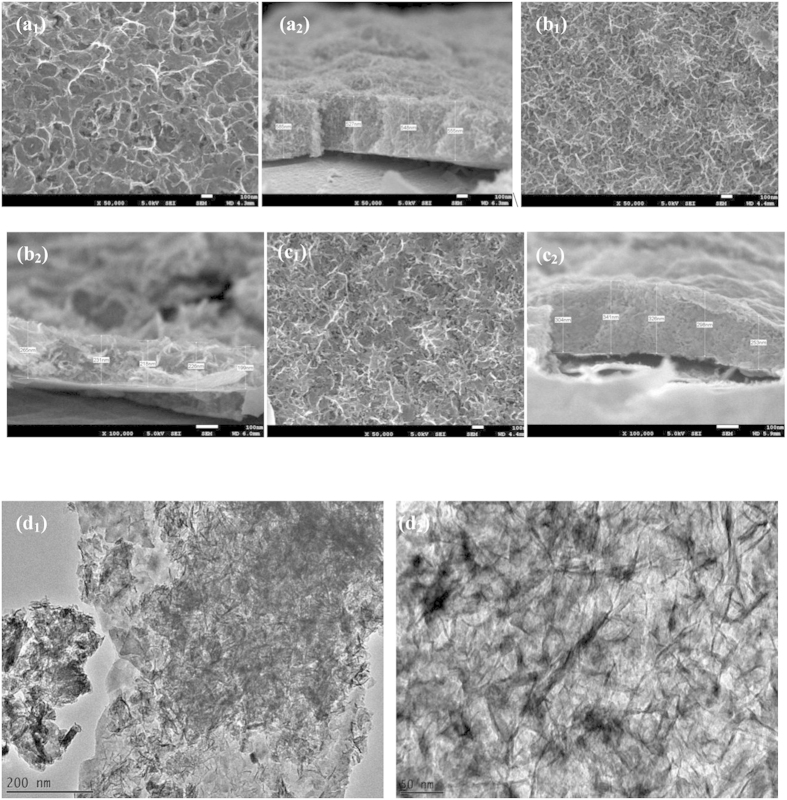
FESEM morphology images and cross section of: (**a_1_, a_2_**) G01, (**b_1_, b_2_**) G03, (**c_1_, c_2_**) G06, and (**d_1_, d_2_**) the TEM images of G03M (left: low magnification; right: high magnification).

The morphological alteration of M30 electrode was obvious when a 0.01 M glucose molecule was added to the electrolyte deposition Fig. 6. The MnO_2_ bunches no longer existed and clearly showed that the porous structure was formed and regularly arranged. The pore diameter was ~60–100 nm when the glucose concentration was 0.01 M, Fig. 6a_1_. The pore diameter decreased dramatically (~30 nm) when the glucose concentration was increased to 0.03 M, Fig. 6b_1_, indicating that the pore structure of rGO/MnO_2_ samples can be tuned by adjusting the glucose concentration in the electrodeposition electrolyte. The average thickness of the G03 electrode was also reduced to 265 nm Fig. 6b_2_. A high number of pores that built from interconnected nanoflakes structures in the G03 electrode is believed to improve the porosity and provide a unique conductive network. This observation might be due to a slower rate of MnO_2_ electrocrystallization, which allows the atoms to arrange themselves at the lowest energy site. According to Babakhani *et al.*, the influential factors, such as the concentration of the deposition electrolyte, would affect the electrocrystallization rate of MnO_2_^28^. Further increases of glucose concentration 0.06 M (G06), deposition rate became slower and lead to flakes not readily growth uniform during the electrodeposition period, Fig. 6c_1_. The TEM of G03 electrode is shown in Fig. 6d_1_. The deposits have a uniform and well-spread rGO sheet, which is covered by MnO_2_ nanoflakes. The interconnected structure, which creates the porous structure, can also be clearly observed at a high magnification of TEM (Fig. 6d_2_. This unique structure has several advantages: (i) the porous structure greatly facilitates the ion diffusion from the electrolyte into the electrode matrix, which promotes the specific capacitance due to high utilization of MnO_2_; (ii) thin deposited materials that are able to shorten the diffusion path of electrons and ions; (iii) the interconnected flakes structure without agglomeration could exhibit the excellent electrochemical performance as an electrode for a supercapacitor^13^.

**Figure 7:**
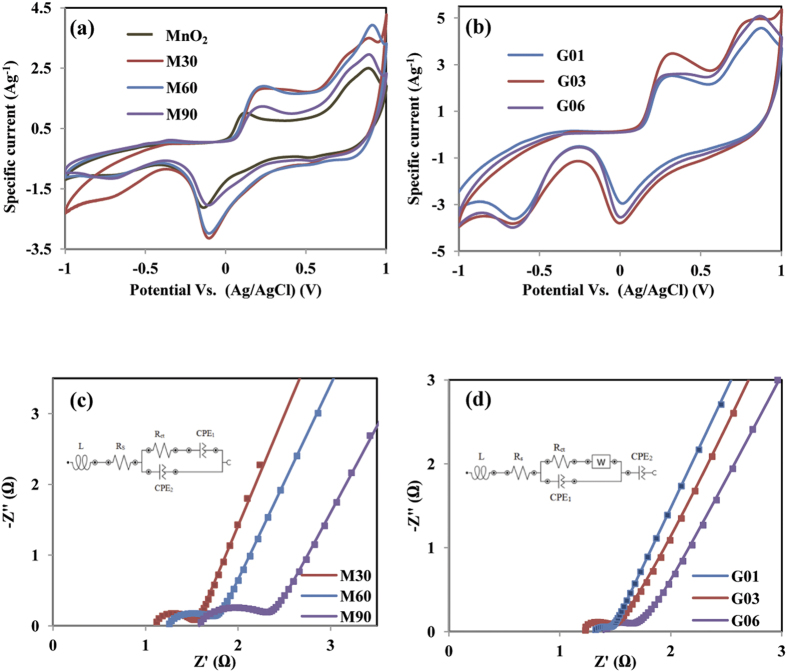
CV curve in 0.5 M Na_2_SO_4_ electrolyte at a scan rate of 5 mVs^**−**1^ of: (**a**) pristine MnO_2_, M30, M60, and M90, (**b**) G01, G03, and G06; Nyquist plot of: (**c**) M30, M60, and M90, (**d**) G01, G03, and G06.

The electrochemical properties of the materials were characterized by cyclic voltammetry (CV) and galvanostatic charge-discharge measurements. CV has been considered to be a suitable technique to investigate the occurrence of faradic or non-faradic reactions in the electrode^29^. The CV curve of pristine MnO_2_, M30, M60, and M90 electrodes in the potential range from −1 V to +1 V at scan rate of 5 mVs^**−**1^ in the 0.5 M Na_2_SO_4_ electrolyte are shown in Fig. 7a. A pair of distinct anodic and cathodic peaks can be clearly observed around 0.2 V and −0.1 V, while other less-intense anodic and cathodic peaks are around 0.9 V/−0.7 V. These peaks are believed to be derived mainly from the redox pairs of Mn^2+^/Mn^3+^. The current response for M30, M60, and M90 is much higher than that of pure MnO_2_, inferring that rGO/MnO_2_ samples have better charge transfer kinetics, due to higher utilization of active Mn species. The calculated specific capacitances from the CV curve for pristine MnO_2_, M30, M60, and M90 electrodes are 167 Fg^**−**1^, 264 Fg^**−**1^, 220 Fg^**−**1^, and 175 Fg^**−**1^, respectively. The highest specific capacitance is found in the M30 electrode, which can be attributed to the combined contribution of redox pseudocapacitance of MnO_2_ and the electrical double layer capacitance of the rGO. The improved performance of the M30 electrode may also be due to a high content of graphene carbon, which is beneficial to shortening the cation path into the electrode matrix and reducing the transfer resistance. CV curve of the deposited G03 electrode at a scan rate of 5 mVs^**−**1^ in a Na_2_SO_4_ electrolyte solution is shown in Fig. 7b. Compared to the CV curve of M30, CV curve of G03 electrode has a similar shape and potential position of anodic and cathodic peaks. However, the current response of G03 is higher, indicating that the effective utilization of the MnO_2_ increased^27^. The calculated specific capacitances are 377 Fg^**−**1^, 430 Fg^**−**1^, and 361 Fg^**−**1^ for G01, G03, and G06 at a scan rate of 5 mVs^**−**1^. Electrode G03 exhibits 63% specific capacitance improvement compared to M30, which is attributed to the uniform morphology structure, less thickness, and low transfer resistance. A further increase of glucose concentration decreased the capacitance to 37% (G06), which might be due to less MnO_2_ available for the reaction.

The characteristic of ion transport resistance for all samples has been investigated by electrochemical impedance spectra (EIS). The Nyquist plot of all deposits electrode in a frequency range of 0.1 Hz until 100 kHz in 0.5 M Na_2_SO_4_ electrolyte is shown in Fig. 7c,d. The equivalent circuit in accordance with Nyquist plots is fitted using Nova software and parameters are shown in Table 2. The intercept of the arc on the *x*-axis at high frequency region is called an equivalent series resistance (R_s_) which represents combination resistance of ionic resistance of electrolyte, contact resistance and internal resistance of the material. This value is almost same for all electrodes within the logical magnitude error. The semicircle region in high frequency region corresponds to charge transfer resistance, R_ct_^30^,^31^,^32^. Overall, G03 electrode has lowest transfer resistance among all samples, indicating G03 has a better electrochemical performance. The incorporation of 0.03 M glucose in M30 electrode leads to improvise the access for intercalation/deintercalation of cation to electrode matrix. The two constant phase elements of CPE_1_ and CPE_2_ in Fig. 7c were used to replace the double layer capacity and Warburg impedance resistance for semi-infinite linear diffusion, respectively^33^. In glucose system, CPE_2_ represents the faradic impedance which is due to redox transition within the electrode, Fig. 7d^34^. As discuss in previous reports^33^,^35^, two constant phase elements can be describe as Z_CPE1_ = [Q(*j*ω)^n1^]^**−**1^ and Z_CPE2_ = [Q(*j*ω)^n2^]^**−**1^ with −1 ≤ n ≤ 1. The component n is correction factor represents the roughness of electrode and it has value ranging from 0 to 1. Pure capacitance yields n = 1, pure resistance yields n = 0, while n = 0.5 represents Warburg impedance. The value of n_1_ ~ 0.8 in G03 indicates that G03 has a nature porous of electrode, in agreement with TEM result.

**Figure 8:**
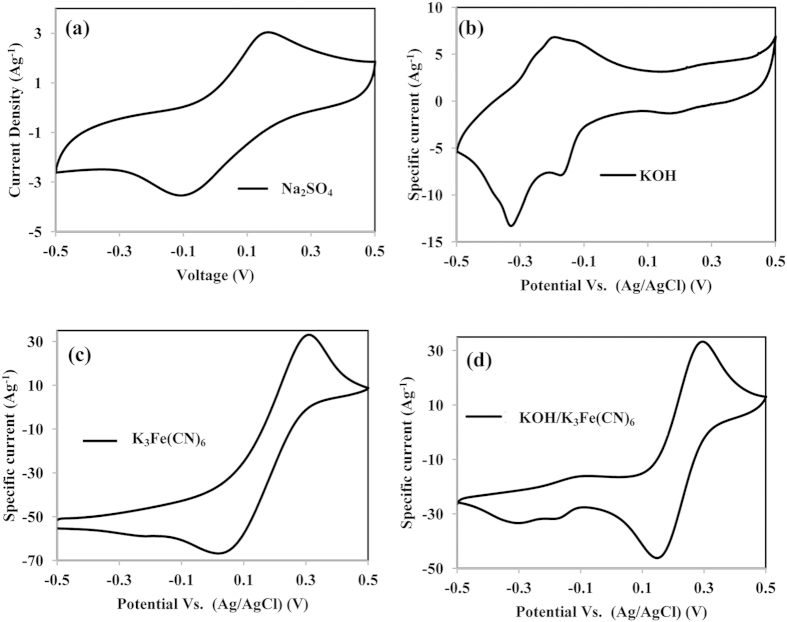
CV curve of G03 at a scan rate of 5 mVs^−1^ within potential scan of −0.5 V to 0.5 V in: (**a**) 0.5 M Na_2_SO_4_, (**b**) 0.5 M KOH, (**c**) 0.04 M K_3_Fe(CN)_6_, and (**d**) 0.5 M KOH/0.04 M K_3_Fe(CN)_6_ electrolyte solution.

In order to get more information on the capacitive performance of the best prepared G03 electrode, G03 electrode was selected to study the performance in three different electrolytes (i.e., 0.5 M Na_2_SO_4_, 0.5 M KOH, and a 0.5 M KOH/0.04 M K_3_Fe(CN)_6_ electrolyte solution. The capacitive performance is believed to be influenced by the size of the cation, cation mobility, and rate of adsorption/desorption at the electrode-electrolyte surface^30^. All CV and charge-discharge performances were studied in a potential range of −0.5 V to 0.5 V. The CV curve of the G03 electrode in three different electrolytes at a scan rate of 5 mVs^**−**1^ is shown in Fig. 8. There is no pitting corrosion of SS substrate found in the potential range of −0.5 to 0.5 V in this three different electrolyte, displayed in supplementary Fig. 1 (supplementary information).

The CV curve of the G03 electrode in 0.5 M Na_2_SO_4_ and 0.5 M KOH electrolyte solutions shown in Fig. 8a,b, the electrode reaction occurred according to Eq. (1, 2)^30^,^36^. The current response in 0.5 M KOH is found to be higher than in the 0.5 M Na_2_SO_4_ electrolyte. This behaviour might be due to the smaller K^+^ size, which can enhance the chemisorption reaction rate, thus optimizing the pseudocapacitance^37^. The specific capacitance of G03 in the Na_2_SO_4_ and KOH electrolyte solutions calculated from the CV curve are 370 Fg^**−**1^ and 804 Fg^**−**1^.


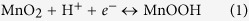



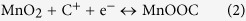


where C is Na^2+^ or K^+^

The best performance of the G03 electrode in KOH was obtained. The redox mediator electrolyte, 0.04 M of K_3_Fe(CN)_6_, was then added into a 0.5 M KOH electrolyte solution with a volume ratio of 1:1. The CV curve in Fig. 8d reveal that the additional of redox mediator has increase the current response drastically. The highest anodic/cathodic peaks at 0.27 V/0.15 V is assign to the charging and discharging process of K_4_Fe(CN)_6_ to K_3_Fe(CN)_6_ (Fig. 8c), which undergoes the reaction shown in Eq. 3^38^, while other less-intensely observed redox peaks originated from electrode reactions with KOH electrolytes. Both instances of redox reactions of rGO/MnO_2_ in KOH and the reaction of K_3_Fe(CN)_6_ in the CV curve indicates that the reactions occur simultaneously and independently^38^. The calculated specific capacitance is 5135 Fg^**−**1^ from the curve, the specific capacitance was found to increase 538% after an addition of 0.04 M K_3_Fe(CN)_6_ electrolyte solution into 0.5 M KOH. In this system, the high capacitance could attribute from the couple of [Fe(CN)_6_]^3**−**^/[Fe(CN)_6_]^4**−**^ in the electrolyte and highly electroactive electrode, Eq. (4). The [Fe(CN)_6_]^3**−**^ will accept the electron via reduction of hexacyanoferrate (III) to (II) when electrode is charging, then the [Fe(CN)_6_]^4**−**^ return to [Fe(CN)_6_]^3**−**^ when reaction is reversible and provide electrons for transition process of Mn(III) to Mn (II). This performance helps the active material to lose and gain electron smoothly and improve the capacitive performance^39^,^40^.


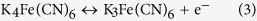



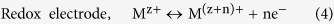


where M is Mn^2+^ cations, and 1 ≤ n ≤ z.

**Figure 9:**
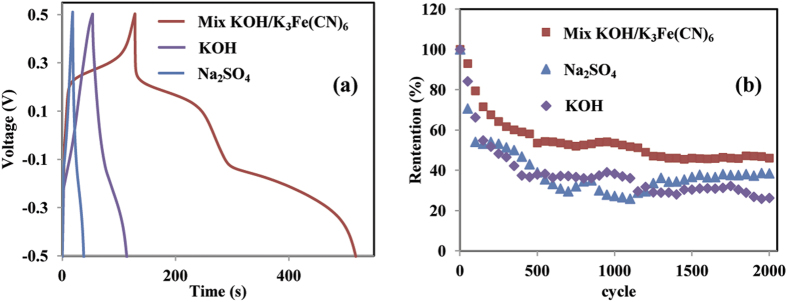
(**a**) Charge-discharge curve of G03, and (**b**) The cyclability test of G03 electrode in three different electrolytes.

Figure 9a displays the charge-discharge (CDC) profile of the G03 electrode in three different electrolytes at current density of 20 Ag^**−**1^. The CDC profile showed a slightly non-linear curve, which indicates the occurrence of a redox reaction within this voltage range^41^. The charge-discharge curve of G03 in the Na_2_SO_4_ and KOH electrolyte solutions has a small plateau potential around −0.1 V in the discharging curve, which corresponds to the reduction of MnO_2_. In the KOH/K_3_FeCN_6_ electrolyte solution, two potential plateaus were found. The first potential at −0.1 V is due to the reduction of MnO_2_, and the second potential at 0.2 V corresponds to the redox reaction of K_4_Fe(CN)_6_. Because both reactions occured, the G03 electrode in the KOH/K_3_Fe(CN)_6_ electrolyte solution at a current density of 20 Ag^**−**1^ has a higher specific capacitance of 13,333 Fg^**−**1^, with a power density of 68.35 kW.kg^**−**1^ and an energy density of 1851 Wh.kg^**−**1^.

The stability test of the 0.03 M electrode in Na_2_SO_4_, KOH, and KOH/ K_3_Fe(CN)_6_ electrolyte solutions was continuously performed in a voltage range from −0.5 V to 0.5 V at a scan rate of 10 mVs^**−**1^ until 2000 cycles, as shown in Fig. 9b. The specific capacitance of G03 in the KOH/ K_3_Fe(CN)_6_ electrolyte solution has retained up to 46% of its initial specific capacitance after 2000^th^ cycle, it has good stability compared to other electrolytes. In comparison with pass studies, rGO/MnO_2_ electrode prepared using hydrothermal and sol gel methods^42^,^43^,^44^ showed good stability but low specific capacitance which is due to high carbon content in the electrode and no obvious redox peaks were observed^42^. In our work, the high capacitance, energy and power density can be assigned to high redox activity of the electrode. The high degradation of the capacitance retention may be due to a high rcurrent pass through the electrode during cyclability test^20^,^30^.

## Discussion

In summary, rGO/MnO_2_ nanocomposites has successfully synthesized by using electrodeposition method. The rGO was found to be clearly covered by MnO_2_ in M30 electrode sample. An ultrathin-deposits electrode with uniform nanoflake structure was obtained after 0.03 M of glucose solution was added into GO/Mn(CH_3_COO)_2_·4H_2_O electrolyte solution. It is believed that the additional 0.03 M glucose solution is a suitable concentration for slowing down the nucleation process of MnO_2_, which results in an excellent flake morphology structure. The depositing of glucose onto SS electrodes was also found to increase the carbon content, which helps to reduce the transfer resistance of cation diffusion paths to the electrode matrix. As a result, the effective use of MnO_2_ resulted in a high specific capacitance of 13,333 Fg^**−**1^ with a power density of 68.35 kW.kg^**−**1^, and an energy density of 1851 Wh.kg^**−**1^ at a current density of 20 Ag-1 in a 0.5 M KOH/ 0.04 M K_3_Fe(CN)_6_ electrolyte solution. Nevertheless, after 2000 cycles at a scan rate of 10 mVs^**−**1^, a retention percentage of 46% was observed. This result may be ascribed to the loss of the active materials in the electrode. The preparation of G03 electrode is a simple, low-cost, and environmentally-friendly method that holds great potential for producing cost-effective and high-energy-density supercapacitors.

## Methods

The MnO_2_-rGO composite was synthesized by a potentiodynamic method on 4 cm^2^ of stainless steel (SS) in the voltage range of 0 to 2 V. The scan rate of 50 mVs^**−**1^ was applied. The electrochemical cell consisted of the steel substrate as the working electrode, a carbon rod as the counter electrode, and Ag/AgCl as the reference electrode. The stainless steel subtracts were sonicated and rinsed with acetone three times and dried at room temperature. Prior to deposition, 50 mg of GO was dispersed in 50 mL of distilled water and ultrasonication treatment for 0.5 h. The *in situ* electrodeposition process was performed in an aqueous solution containing a fixed volume of GO (10 mL) and different volumes (30 mL, 60 mL, and 90 mL) of 0.01 M manganese acetate aqueous solution. The obtained electrodes were dried at a temperature of 300 °C for 6 h and allowed to cool to room temperature before the characterisation step. The prepared samples were denoted as M30, M60, and M90, respectively. The MnO_2_-rGO-glucose carbon composite was prepared by the same aforementioned procedure. In a typical experiment, the deposition electrolytes were prepared by dissolving 0.01 M, 0.03 M, 0.06 M of glucose in 30 mL of distilled water under magnetic stirring. These solutions were then separately mixed with 30 mL manganese acetate (0.01 M) aqueous solution. Consequently, 10 mL GO solution was added drop-wise in the solution and the mixture was sonicated for 22 mins at room temperature. The prepared samples after heating treatment are labeled as G01, G03, and G06.

The X-ray diffraction (XRD) patterns of deposited samples on SS and the powders of deposits were obtained using a PANalytical Empyrean and D8 Advance X-Ray diffractometer-Bruker AXS with CuK_α_ monochromatized radiation at 40 kV and 40 mA. The Raman spectra were obtained using Renishaw inVia Raman microscope with a green beam. The FESEM and TEM images of the electrodes were captured using a Jeol JSM 7600 and Jeol JEM 2100F, respectively. The Energy Dispersive X-ray (EDX) spectrum was collected using an Oxford Instruments apparatus for the elemental analysis of the electrode sample. Thermal gravimetric analysis (TGA) of glucose was performed using a TA Instrument, Q500 with heating rate of 0.01 °C min^**−**1^. The electrochemical test of charge/discharge (CD), cyclic voltammetry (CV), and electrical impedance spectroscopy (EIS) were conducted using potentiostat (Autolab, PGSTAT30). The platinum wire was used as a counter and Ag/AgCl was used for reference electrodes.

## Additional Information

**How to cite this article**: Rusi and Majid S. R. Green synthesis of *in situ* electrodeposited rGO/MnO_2_ nanocomposite for high energy density supercapacitors. *Sci. Rep*. **5**, 16195; doi: 10.1038/srep16195 (2015).

## Supplementary Material

Supplementary Information

## Figures and Tables

**Figure 1:**
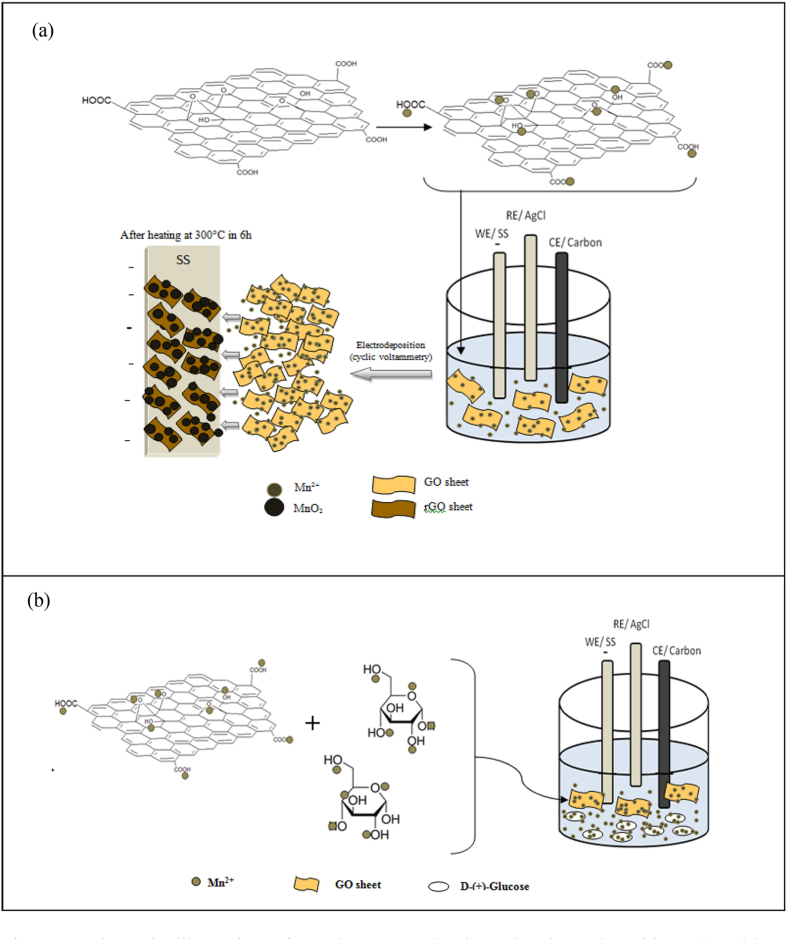
A schematic illustration of rGO/MnO_2_ mechanism via electrodeposition: (**a**) without glucose and (**b**) with glucose.

**Table 1:** The Raman bands, I_Dx_/I_G_ ratio and vibration modes of M30 and G03 electrode.

Bands	Raman shift (cm^−1^)		Ratio (I_Dx_/I_G_)		Vibration mode
	M30	G03	M30	G03	
G	1573.76	1547.81	—	—	Ideal graphitic lattice (E_2g_-symmetry)
D1	1294.93	1315.29	3.09	11.49	Disordered graphitic lattice (graphene layer edges, A_1g_-symmetry)
D2	1617.56	1600.88	1.26	0.48	Disordered graphitic lattice (surface graphene layers, E_2g_-symmetry)
D3	1529.42	1517.35	1.57	0.36	Amorphous carbon (Gaussian or Lorentzian line shape)
D4	1202.22	1196.59	7.07	8.16	Disordered graphitic lattice (A_1g_-symmetry), polyenes, ionic impurities

**Table 2:** The Equivalent circuit parameters deducted by fitting Nyquist plots.

Sample	L (×10^−7^H)	R_s_ (Ω)	R_ct_ (Ω)	CPE_1_ (×10^−3^ Ω)^−1^	CPE_2_ (×10^−3^ Ω)^−1^	n_1_	n_2_
M30	5.23	1.01	0.55	0.41	50.50	0.78	0.81
M60	8.98	1.10	0.71	2.98	47.40	0.62	0.78
M90	8.33	1.48	0.92	1.89	56.00	0.67	0.76
G01	9.98	1.10	0.40	1.08	35.60	0.64	0.78
G03	3.14	1.10	0.32	0.39	32.5	0.81	0.83
G06	3.79	1.30	0.36	0.56	28.3	0.76	0.85
