# Supplementary material for: Green synthesis of in situ electrodeposited rGO/MnO2 nanocomposite for high energy density supercapacitors
Source: Sci Rep. 2015 Nov 5;5:16195. doi: 10.1038/srep16195 (PMC4633667; doi:10.1038/srep16195)
Supplement: Supplementary Information [file srep16195-s1.pdf]

Supplementary Information

Green synthesis of in situ electrodeposited rGO/MnO_2 nanocomposite for high energy density supercapacitors

Rusi & S.R. Majid

Centre for Ionics University of Malaya, Department of Physics, Faculty of Science, University of Malaya, 50603 Kuala Lumpur, Malaysia.

*E-mail: shana@um.edu.my

1. Supplementary Figures.

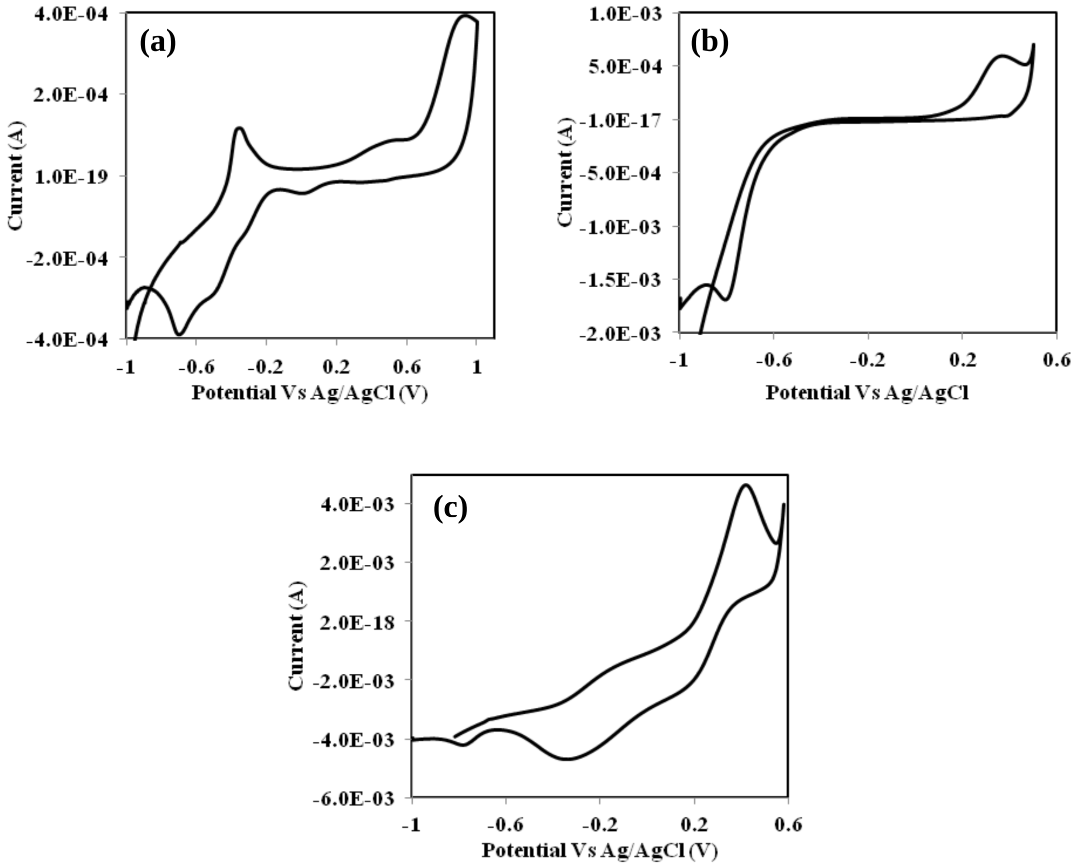

Supplementary Figure 1 | The cyclic voltammetry of plain stainless steel in: (a) Na_2SO_4, (b) KOH and (c) mix KOH/$K_3Fe(CN)_6$.

The Cyclic voltammetry (CV) curves show different shapes compared to the CV curve of deposited rGO/MnO_2 on top of SS (Fig.8). The redox peaks in this figure are not present in deposited rGO/MnO_2 on SS electrode, indicating there is no reaction of SS involve in redox reaction of rGO/MnO_2. Additionally, there is no pitting potential found in three different electrolytes within the potential ranging from -0.5 V to 0.5 V indicate there is no corrosion process has taken place [1].

Supplementary Reference

1. Freire, L. *et al*. Electrochemical and analytical investigation of passive films formed on stainless steels in alkaline media. *Cem. Concr. Compos*. **34**, 1075-1081 (2012).
